# Supplementary material for: ATP-free in vitro biotransformation of starch-derived maltodextrin into poly-3-hydroxybutyrate via acetyl-CoA
Source: Nat Commun. 2024 Apr 16;15:3267. doi: 10.1038/s41467-024-46871-y (PMC11021460; doi:10.1038/s41467-024-46871-y)

Supplementary Information

ATP-free in vitro biotransformation of starch-derived maltodextrin into poly-3-hydroxybutyrate via acetyl-CoA

Xinlei Wei[1,2], Xue Yang[2], Congcong Hu[1,2,3], Qiangzi Li[1,2,4], Qianqian Liu[2], Yue Wu[2], Leipeng Xie[1,2], Xiao Ning[1,2,4], Fei Li[1,2], Tao Cai[2], Zhiguang Zhu[1,2,4,5], Yi-Heng P. Job Zhang[1,2,4,5], Yanfei Zhang[2,4,5], Xuejun Chen[2], and Chun You[1,2,4,5]*

[1] In vitro Synthetic Biology Center, Tianjin Institute of Industrial Biotechnology, Chinese Academy of Sciences, 32 West 7th Avenue, Tianjin Airport Economic Area, Tianjin 300308, People's Republic of China

[2] Tianjin Institute of Industrial Biotechnology, Chinese Academy of Sciences, 32 West 7th Avenue, Tianjin Airport Economic Area, Tianjin 300308, People's Republic of China

[3] Key Laboratory of Industrial Fermentation Microbiology, Ministry of Education, Tianjin Industrial Microbiology Key Laboratory, College of Biotechnology, Tianjin University of Science and Technology, Tianjin 300457, People's Republic of China

[4] University of Chinese Academy of Sciences, 19A Yuquan Road, Shijingshan District, Beijing 100049, People's Republic of China

[5] National Technology Innovation Center of Synthetic Biology, Tianjin 300308, People's Republic of China

*Corresponding author: Chun You, Phone: (+86)-022-24828795, Email: you_c@tib.cas.cn

This PDF file includes

Supplementary Fig. 1 | SDS-PAGE analysis of purified enzymes used in the designed ivSEB. 3

Supplementary Fig. 2 | Gas chromatographic profiles of methyl esters from PHB standards. 4

Supplementary Fig. 3 | Production of PHB from 100 mM maltodextrin by the designed ivSEB. 5

Supplementary Fig. 4 | Effect of $NADP^+$ input concentrations on the production of PHB. 6

Supplementary Fig. 5 | Effect of CoA input concentrations on the production of PHB. 7

Supplementary Fig. 6 | Effect of TPP input concentrations on the production of PHB. 8

Supplementary Fig. 7 | Comparisons of results predicted by the kinetic models with the experimental data before and after the first round of model fitting. 9

Supplementary Fig. 8 | Simulation optimization of enzyme concentrations using Model 1. 10

Supplementary Fig. 9 | A comparison of simulation results with the experimental data at optimized enzyme concentrations predicted by the first round of simulation optimization using Model 1. 12

Supplementary Fig. 10 | Simulation optimization of enzyme concentrations using Model 2. 13

Supplementary Fig. 11 | Production of PHB from edible crude starch. 15

Supplementary Fig. 12 | Parameter scan of V_{max} of PhaC using Model 2 for the consumption of 100 mM substrate. 16

Supplementary Fig. 13 | Experimental optimization of the loading amounts of 4GT and PPGK. .. 17

Supplementary Fig. 14 | Production of PHB from 200 mM glucose equivalent of maltodextrin without doubling the enzyme concentrations. 18

Supplementary Table 1. Information of enzymes used in the designed ivSEB 19

Supplementary Table 2. Equilibrium constant (k_{eq}) and Gibbs free energy change ($\Delta_r G'^{\circ}$) values of each enzymatic reaction in the designed ivSEB 21

Supplementary Table 3. Kinetic functions for COPASI modeling 22

Supplementary Table 4. Stoichiometric coefficients of enzymatic reactions of the designed ivSEB 23

Supplementary Table 5. Kinetic parameters for COPASI modeling 24

Supplementary Table 6. Comparison of enzyme loading amounts prior and after in silico optimization 27

Supplementary Table 7. Molecular weights of PHB samples 28

Supplementary Table 8. Primers used for plasmid construction 29

Supplementary References... 30

Source Data file: Supplementary Fig. 1 32

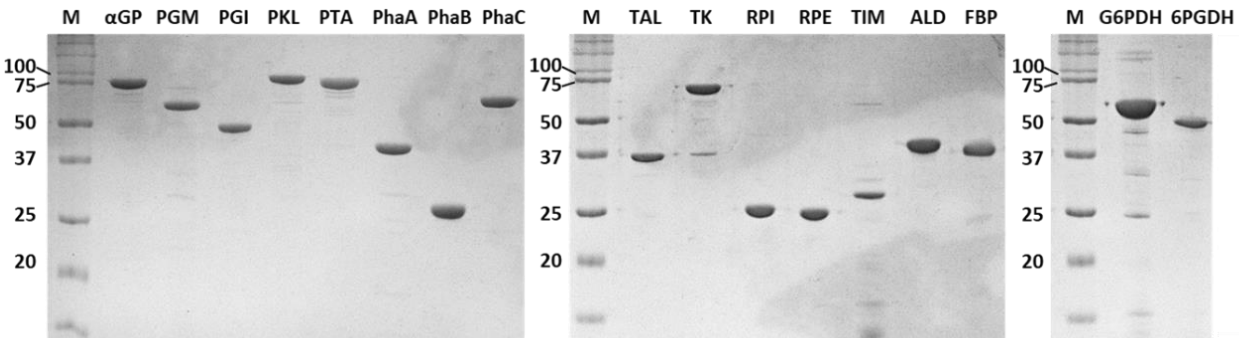

Supplementary Fig. 1 | SDS-PAGE analysis of purified enzymes used in the designed ivSEB. M, molecular weight markers with the sizes shown aside in kDa; αGP, α-glucan phosphorylase; PGM, phosphoglucomutase; PGI, phosphoglucose isomerase; PKL, phosphoketolase; PTA, phosphate acetyltransferase; PhaA, acetyl-CoA acetyltransferase; PhaB, acetoacetyl-CoA reductase; PhaC, PHB synthase; TAL, transaldolase; TK, transketolase; RPI, ribose 5-phosphate isomerase; RPE, ribose 5-phosphate 3-epimerase; TIM, triose phosphate isomerase; ALD, fructose-bisphophate aldolase; FBP, fructose 1,6-bisphosphatase; G6PDH, glucose 6-phosphate dehydrogenase; 6PGDH, 6-phosphogluconate dehydrogenase.

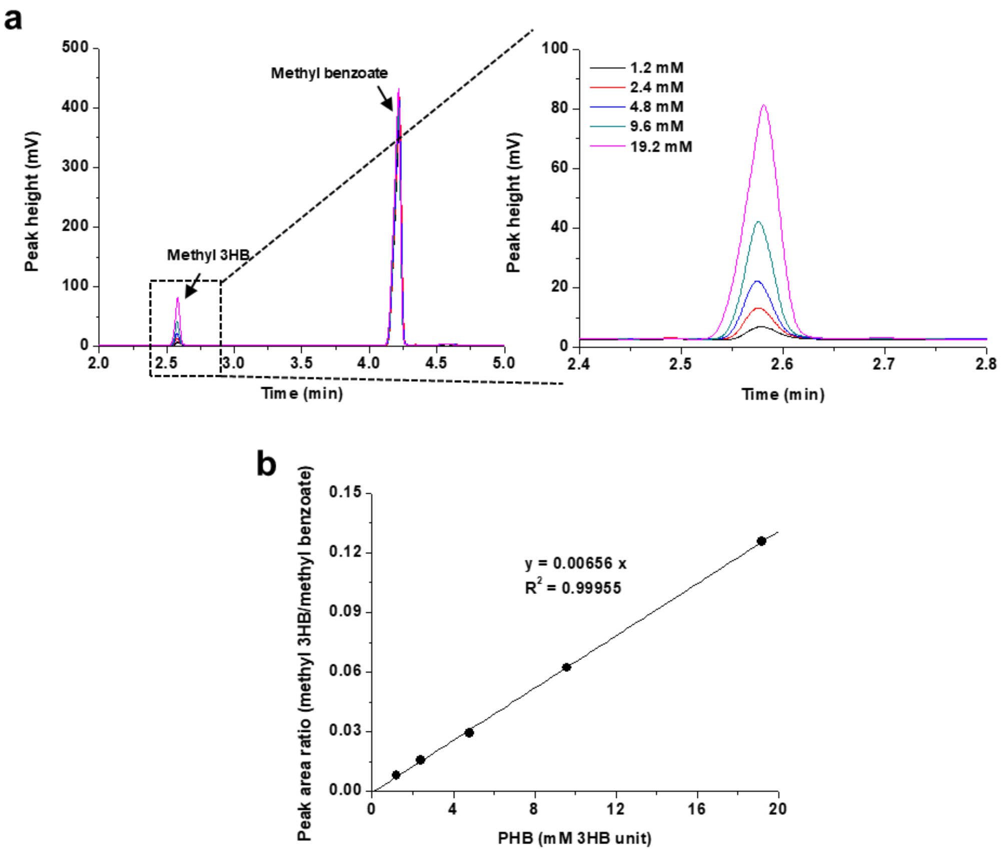

Supplementary Fig. 2 | Gas chromatographic profiles of methyl esters from PHB standards. a, Gas chromatogram showing profiles of methyl esters resulting from acidic methanolysis of PHB standards (yielding methyl 3HB) and benzoic acid (yielding methyl benzoate). The latter was employed as an internal standard for PHB quantification. **b**, Standard curve of PHB plotted based on the gas chromatographic results. Source data are provided as a Source Data file.

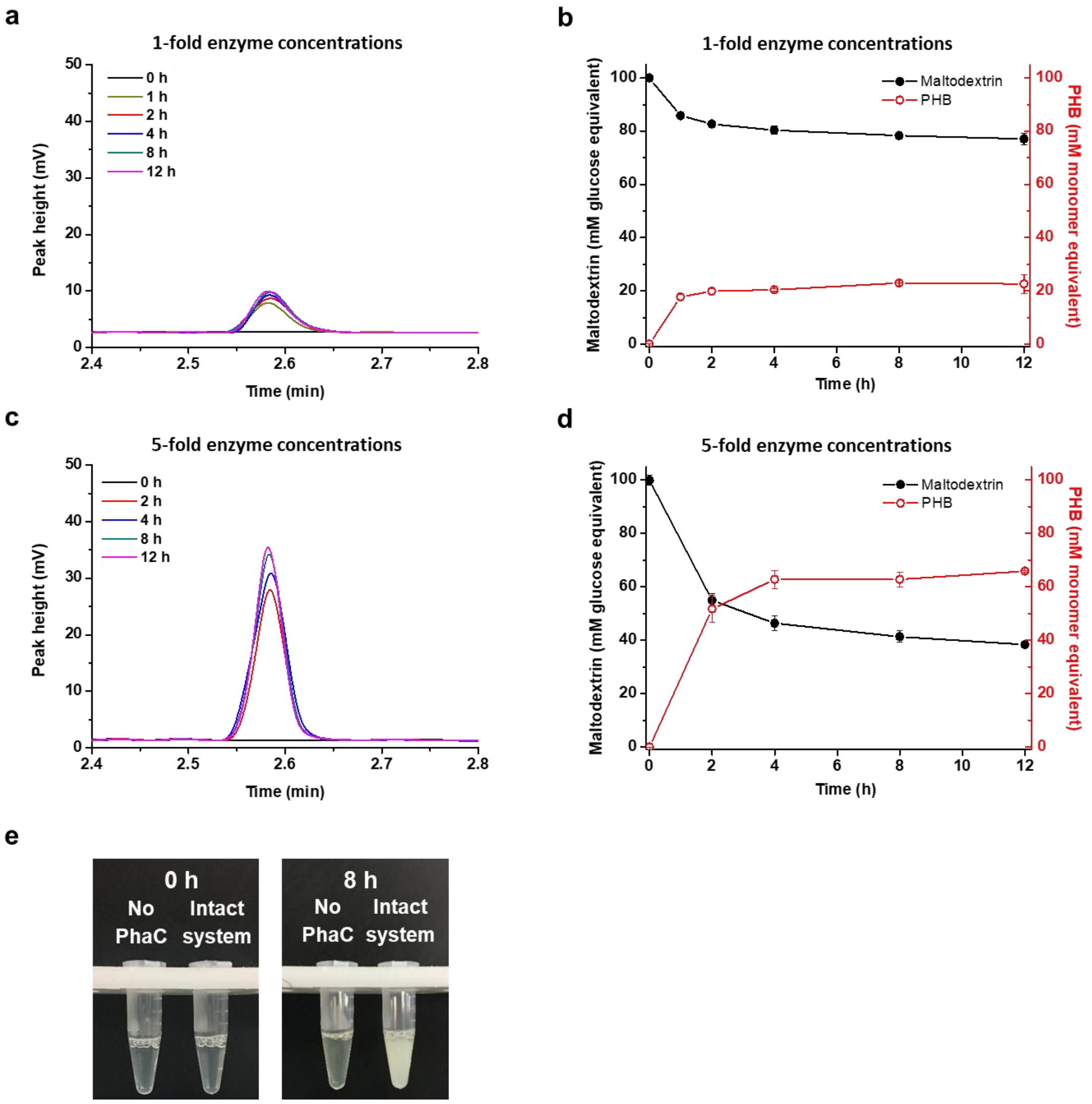

Supplementary Fig. 3 | Production of PHB from 100 mM maltodextrin by the designed ivSEB. a and **b**, Each enzyme was loaded at 1 U/mL except that PhaA was loaded at 1 mg/mL (i.e. 1-fold enzyme concentrations). **c**, **d** and **e**, Each enzyme was loaded at 5 U/mL except that PhaA was loaded at 5 mg/mL (i.e. 5-fold enzyme concentrations). **a** and **c**, Gas chromatographic profiles of methanolyzed PHB samples produced from 100 mM IA-treated maltodextrin. **b** and **d**, Concentrations profiles of maltodextrin and PHB. Reactions were performed in triplicate (n = 3 biologically independent samples) and data are presented as mean values ± standard deviation (SD). Source data are provided as a Source Data file. **e**, Photos showing the reaction samples of **Supplementary Fig. 3d**.

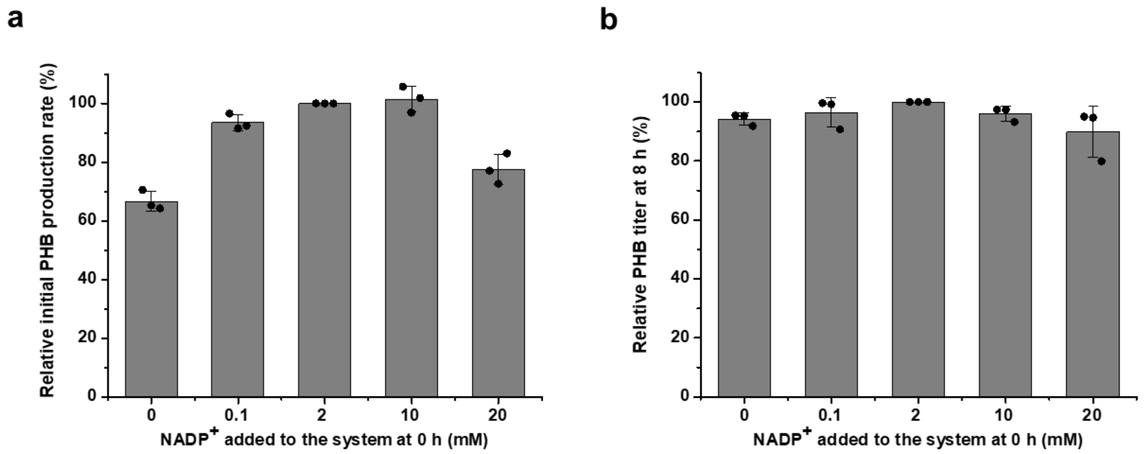

Supplementary Fig. 4 | Effect of $NADP^+$ input concentrations on the production of PHB. a, Effect of $NADP^+$ input concentration on initial PHB production rate (defined as the amount of PHB produced within the first hour). **b**, Effect of $NADP^+$ input concentration on PHB titer at 8 h. The reactions were carried out at 37 °C in 200 mM Tris-HCl buffer (pH 7.4) containing 10 mM $MgCl_2$, 0.5 mM $MnCl_2$, 10 μg/mL ampicillin, 5 μg/mL kanamycin, 10 mM sodium phosphate (pH 7.4), 0.5 mM TPP, 0.5 mM CoA, 100 mM IA-debranched maltodextrin, and enzymes. Each enzyme was loaded at 5 U/mL except that PhaA was loaded at 5 mg/mL (approximately 3.9 mU/mL). Additional $NADP^+$ were supplemented to the samples at 0 h, with their concentrations in the reaction mixture indicated on the x axes. Relative initial PHB production rates and PHB titers at 8 h were calculated using the results obtained at 2 mM $NADP^+$ as 100%. Reactions were performed in triplicate (n = 3 biologically independent samples) and data are presented as mean values ± SD. Source data are provided as a Source Data file.

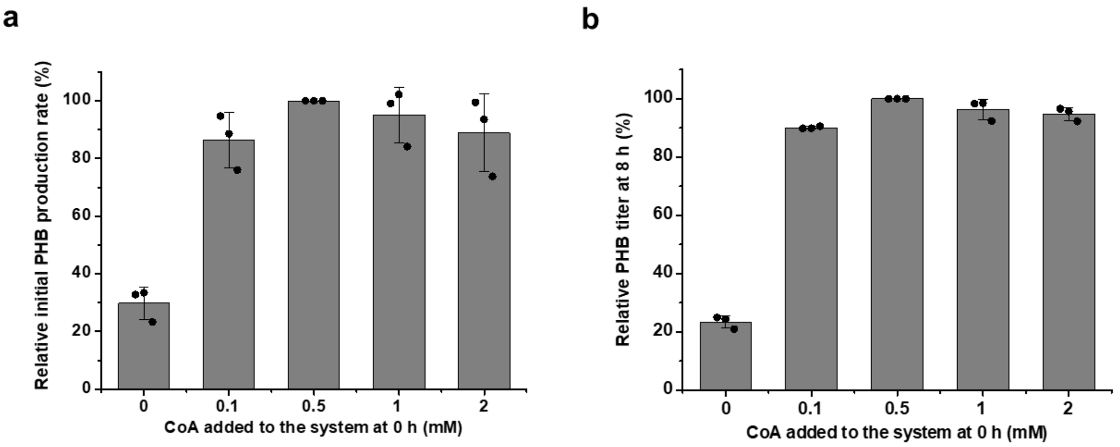

Supplementary Fig. 5 | Effect of CoA input concentrations on the production of PHB. a, Effect of CoA input concentration on initial PHB production rate (defined as the amount of PHB produced within the first hour). **b**, Effect of CoA input concentration on PHB titer at 8 h. The reactions were carried out at 37 °C in 200 mM Tris-HCl buffer (pH 7.4) containing 10 mM $MgCl_2$, 0.5 mM $MnCl_2$, 10 μg/mL ampicillin, 5 μg/mL kanamycin, 10 mM sodium phosphate (pH 7.4), 2 mM $NADP^+$, 0.5 mM TPP, 100 mM IA-debranched maltodextrin, and enzymes. Each enzyme was loaded at 5 U/mL except that PhaA was loaded at 5 mg/mL (approximately 3.9 mU/mL). Additional CoA were supplemented to the samples at 0 h, with their concentrations in the reaction mixture indicated on the x axes. Relative initial PHB production rates and PHB titers at 8 h were calculated using the results obtained at 0.5 mM CoA as 100%. Reactions were performed in triplicate (n = 3 biologically independent samples) and data are presented as mean values ± SD. Source data are provided as a Source Data file.

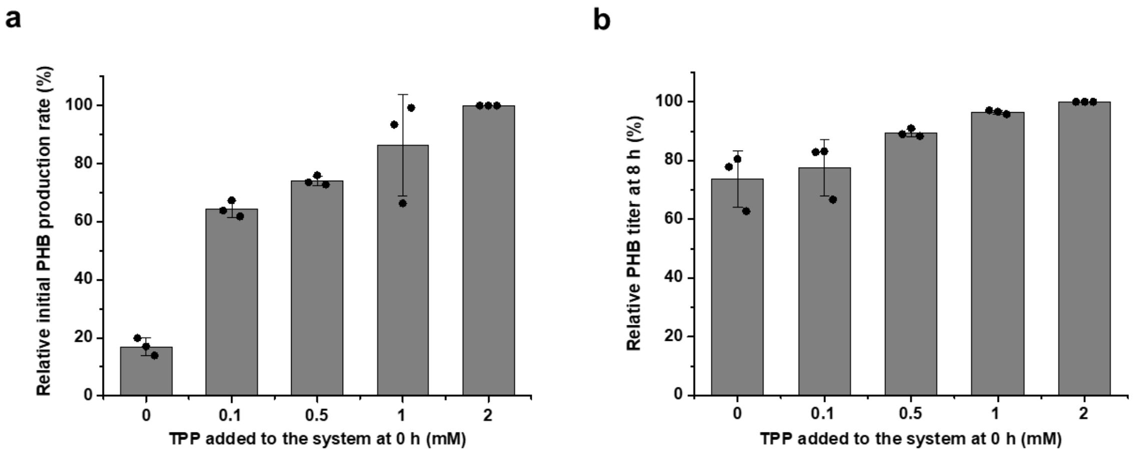

Supplementary Fig. 6 | Effect of TPP input concentrations on the production of PHB. a, Effect of TPP input concentration on initial PHB production rate (defined as the amount of PHB produced within the first hour). **b**, Effect of TPP input concentration on PHB titer at 8 h. The reactions were carried out at 37 °C in 200 mM Tris-HCl buffer (pH 7.4) containing 10 mM $MgCl_2$, 0.5 mM $MnCl_2$, 10 μg/mL ampicillin, 5 μg/mL kanamycin, 10 mM sodium phosphate (pH 7.4), 2 mM $NADP^+$, 0.5 mM CoA, 100 mM IA-debranched maltodextrin, and enzymes. Each enzyme was loaded at 5 U/mL except that PhaA was loaded at 5 mg/mL (approximately 3.9 mU/mL). Additional TPP were supplemented to the samples at 0 h, with their concentrations in the reaction mixture indicated on the x axes. Relative initial PHB production rates and PHB titers at 8 h were calculated using the results obtained at 2 mM TPP as 100%. Reactions were performed in triplicate (n = 3 biologically independent samples) and data are presented as mean values ± SD. Source data are provided as a Source Data file.

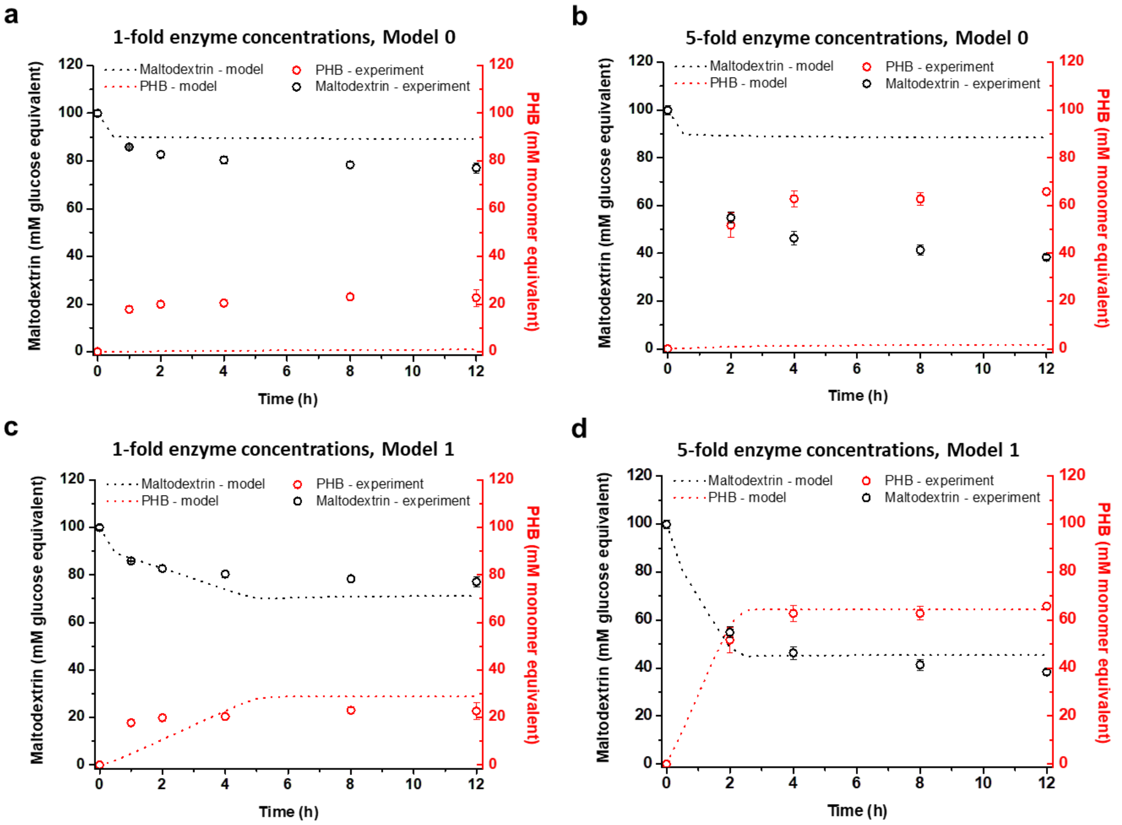

Supplementary Fig. 7 | Comparisons of results predicted by the kinetic models with the experimental data before and after the first round of model fitting. 100 mM glucose equivalent of IA-treated maltodextrin was used as substrate. **a** and **b**, Comparisons of simulation data obtained from the unfitted Model 0 with experimental results. **c** and **d**, Comparisons of simulation data obtained from Model 1 (which is the model after the first round of model fitting) with experimental results. For **a** and **c**, V_{max} of each enzyme in the model was set as 1 mM/min (except that FPK had a V_{max} of 0.12 mM/min), and each enzyme was loaded at 1 U/mL (except that PhaA was loaded at 1 mg/mL or approximately 0.78 mU/mL) for the experiment. For **b** and **d**, V_{max} of each enzyme in the model was set as 5 mM/min (except that FPK had a V_{max} of 0.6 mM/min), and each enzyme was loaded at 5 U/mL (except that PhaA was loaded at 5 mg/mL or approximately 3.9 mU/mL) for the experiment. Model was constructed as described in the Methods. Kinetic functions and parameters used for model construction refer to **Supplementary Table 2**, **Supplementary Table 3**, and **Supplementary Table 5**. Experimental reactions were performed in triplicate (n = 3 biologically independent samples) and data are presented as mean values ± SD. Source data are provided as a Source Data file.

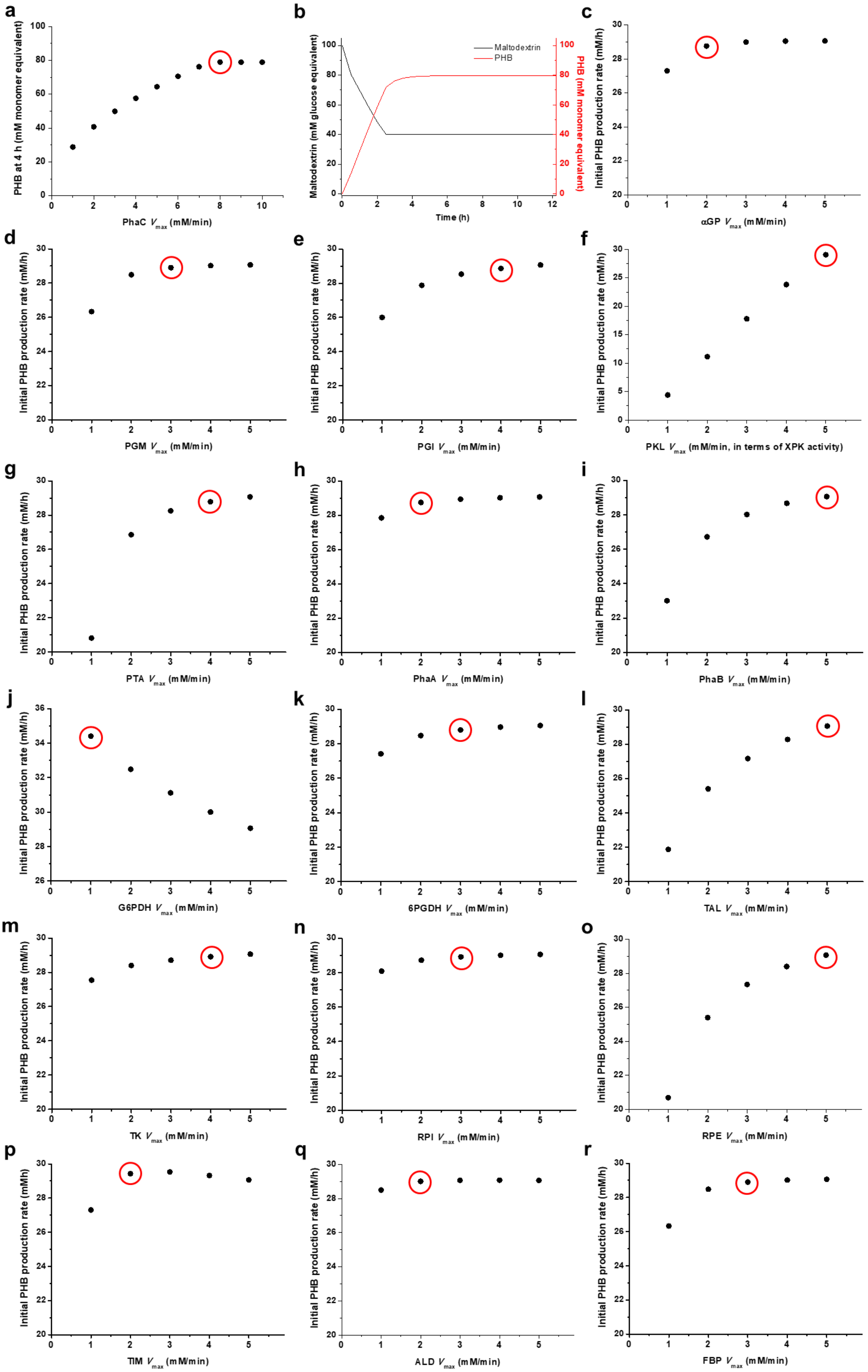
a
PHB at 4 h (mM monomer equivalent)
PhaC V_{max} (mM/min)
b
Maltodextrin (mM glucose equivalent)
PHB (mM monomer equivalent)
Time (h)
Maltodextrin
PHB
c
Initial PHB production rate (mM/h)
αGP V_{max} (mM/min)
d
PGM V_{max} (mM/min)
e
PGI V_{max} (mM/min)
f
PKL V_{max} (mM/min, in terms of XPK activity)
g
PTA V_{max} (mM/min)
h
PhaA V_{max} (mM/min)
i
PhaB V_{max} (mM/min)
j
G6PDH V_{max} (mM/min)
k
6PGDH V_{max} (mM/min)
l
TAL V_{max} (mM/min)
m
TK V_{max} (mM/min)
n
RPI V_{max} (mM/min)
o
RPE V_{max} (mM/min)
p
Initial PHB production rate (mMh)
TIM V_{max} (mM/min)
q
ALD V_{max} (mM/min)
r
FBP V_{max} (mM/min)

Supplementary Fig. 8 | Simulation optimization of enzyme concentrations using Model 1. a, Parameter scan of PhaC V_{max}. Optimal V_{max} value of PhaC, highlighted by a red circle, was defined as the minimal V_{max} value that resulted in no less than 99% of the maximal PHB titer at 4 h achieved within the range of V_{max} for scanning. **b**, Model-predicted time-course profiles of maltodextrin and PHB when V_{max} of PhaC was set as 8 mM/min. **c-r**, Parameter scan of V_{max} of the rest of enzymes. **c**, αGP. **d**, PGM. **e**, PGI. **f**, PKL in terms of XPK activity. **g**, PTA. **h**, PhaA. **i**, PhaB. **j**, G6PDH. **k**, 6PGDH. **l**, TAL. **m**, TK in terms of TK-1 activity. **n**, RPI. **o**, RPE. **p**, TIM. **q**, ALD. **r**, FBP. Initial PHB production rate was defined as the amount of PHB produced within the first hour of simulation reaction. Optimal V_{max} value for each enzyme, highlighted by a red circle, was defined as the minimal V_{max} value that results in no less than 99% of the highest initial PHB production rate achieved within the range of V_{max} for scanning. Details of model construction and parameter scan method are described in Methods. Source data are provided as a Source Data file.

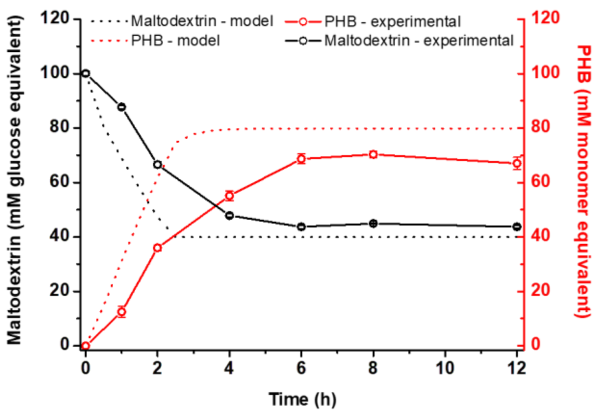

Supplementary Fig. 9 | A comparison of simulation results with the experimental data at optimized enzyme concentrations predicted by the first round of simulation optimization using Model 1. Experimental results are displayed as solid line with symbols, while simulation results were shown as dotted lines. Enzyme loading concentrations were summarized in **Supplementary Table 6**. Experimental reactions were performed in triplicate (n = 3 biologically independent samples) and data are presented as mean values ± SD. Source data are provided as a Source Data file.

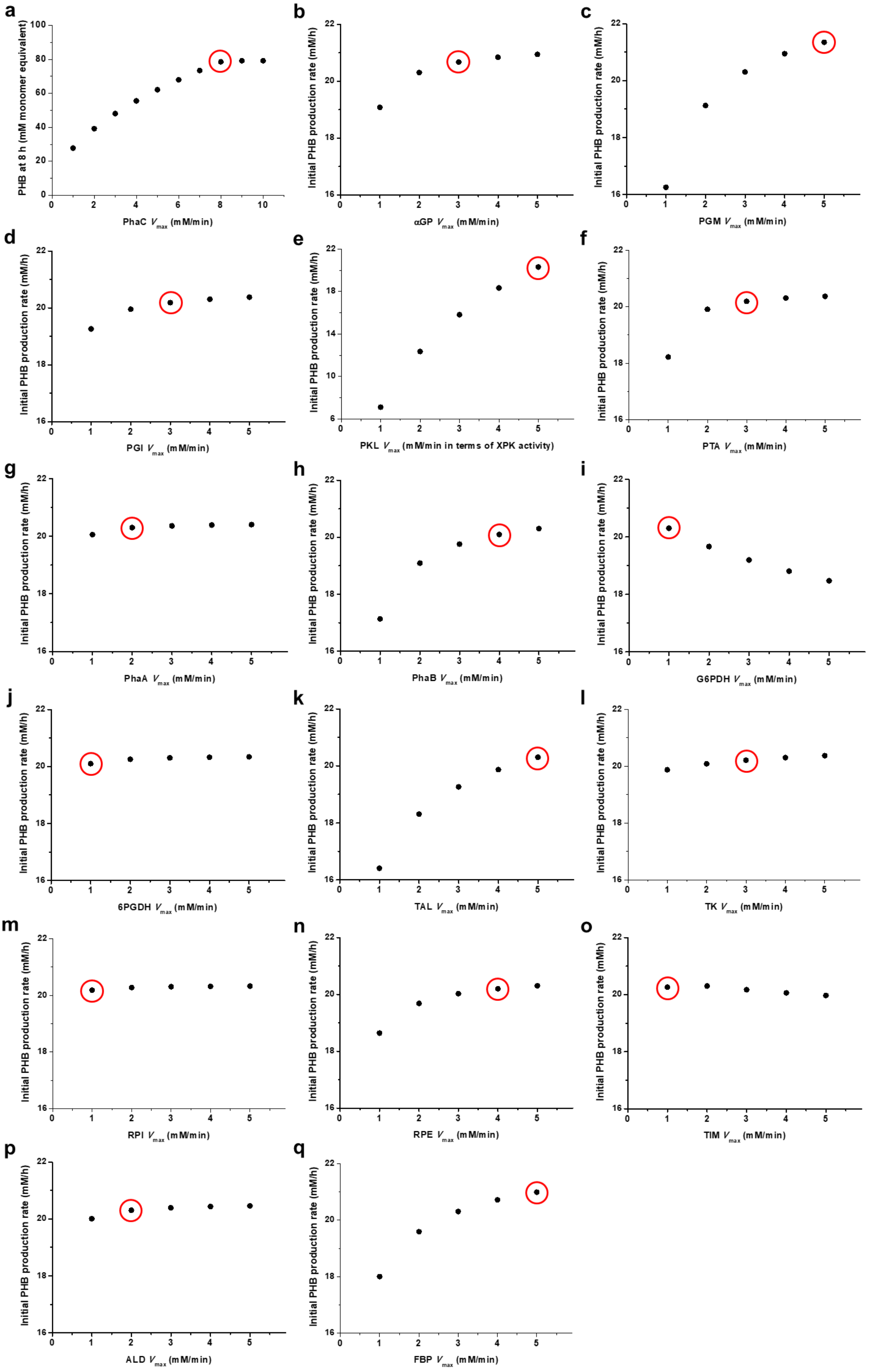
a
PHB at 8 h (mM monomer equivalent)
PhaC Vmax (mM/min)
b
Initial PHB production rate (mM/h)
αGP Vmax (mM/min)
c
PGM Vmax (mM/min)
d
PGI Vmax (mM/min)
e
PKL Vmax (mM/min in terms of XPK activity)
f
PTA Vmax (mM/min)
g
PhaA Vmax (mM/min)
h
PhaB Vmax (mM/min)
i
G6PDH Vmax (mM/min)
j
6PGDH Vmax (mM/min)
k
TAL Vmax (mM/min)
l
TK Vmax (mM/min)
m
RPI Vmax (mM/min)
n
RPE Vmax (mM/min)
o
TIM Vmax (mM/min)
p
ALD Vmax (mM/min)
q
FBP Vmax (mM/min)

Supplementary Fig. 10 | Simulation optimization of enzyme concentrations using Model 2. a, Parameter scan of PhaC V_{max}. Optimal V_{max} value of PhaC, highlighted by a red circle, was defined as the minimal V_{max} value that resulted in no less than 99% of the maximal PHB titer at 8 h achieved within the range of V_{max} for scanning. **b-q**, Parameter scan of V_{max} of the rest of enzymes. **b**, αGP. **c**, PGM. **d**, PGI. **e**, PKL in terms of XPK activity. **f**, PTA. **g**, PhaA. **h**, PhaB. **i**, G6PDH. **j**, 6PGDH. **k**, TAL. **l**, TK in terms of TK-1 activity. **m**, RPI. **n**, RPE. **o**, TIM. **p**, ALD. **q**, FBP. Initial PHB production rate was defined as the amount of PHB produced within the first hour of simulation reaction. Optimal V_{max} value for each enzyme, highlighted by a red circle, was defined as the minimal V_{max} value that results in no less than 99% of the highest initial PHB production rate achieved within the range of V_{max} for scanning. Details of model construction and parameter scan method are described in Methods. Source data are provided as a Source Data file.

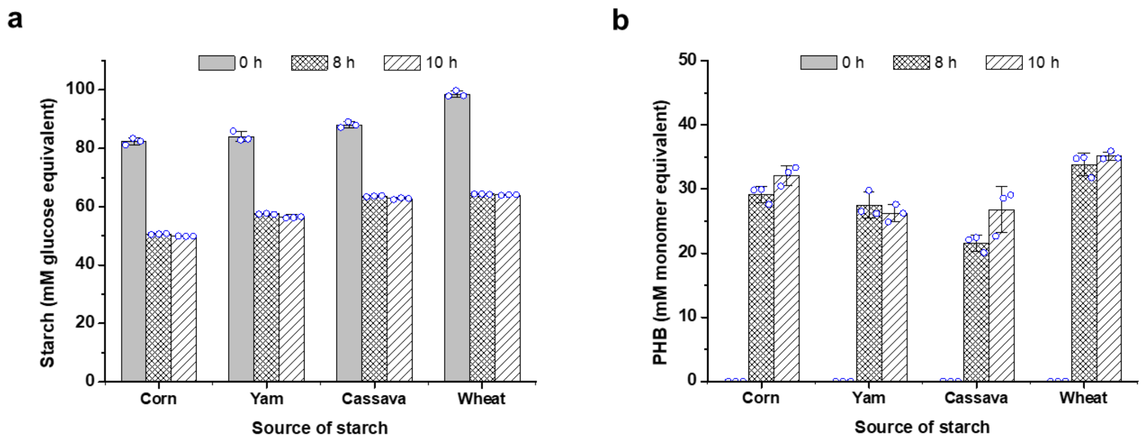

Supplementary Fig. 11 | Production of PHB from edible crude starch. a, Amount of residual starch in the reaction solutions after reaction for 0, 8, and 10 h. **b**, Amount of PHB in the reaction solutions after reaction for 0, 8, and 10 h. Reactions were performed in triplicate (n = 3 biologically independent samples) and data are presented as mean values ± SD. Source data are provided as a Source Data file.

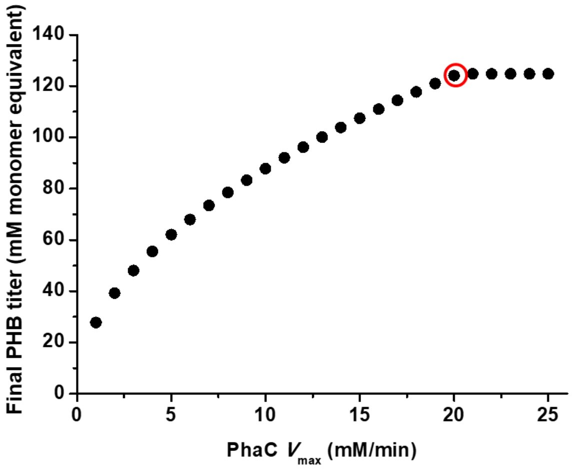

Supplementary Fig. 12 | Parameter scan of V_{max} of PhaC using Model 2 for the consumption of 100 mM substrate. For this task, the previously set "Event" that only up to 60% of the 100 mM maltodextrin could be consumed (see Methods) was deleted. The final PHB titer, defined as the PHB titer at 30 h, was investigated under a PhaC V_{max} range of 1 – 25 mM/min. The predicted optimal V_{max} of PhaC was highlighted by a red circle. Source data are provided as a Source Data file.

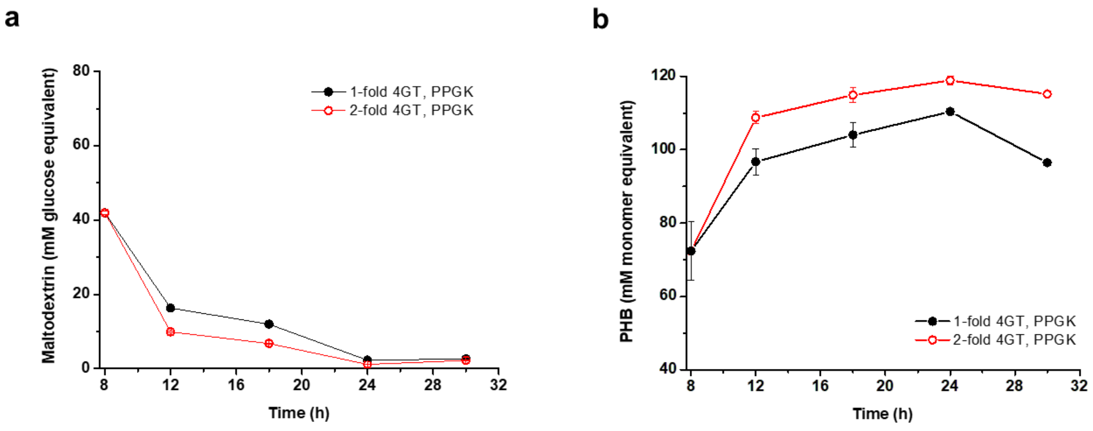

Supplementary Fig. 13 | Experimental optimization of the loading amounts of 4GT and PPGK. **a**, Concentrations of residual maltodextrin in the reaction solutions. **b**, Concentrations of PHB in the reaction solutions. 100 mM IA-debranched maltodextrin was used as the substrate. The reaction was initiated under the same conditions as that in **Fig. 4d**, except that the concentration of PhaC was raised to 20 U/mL. At 8 h, 4GT, and PPGK were added to the reaction mixture at either 1-fold concentrations (0.1 U/mL or approximately 0.33 mg/mL 4GT, 1.0 U/mL or approximately 0.01 mg/mL PPGK) or 2-fold concentrations (0.2 U/mL or approximately 0.67 mg/mL 4GT, 2.0 U/mL or approximately 0.02 mg/mL PPGK) together with 20 mM sodium hexametaphosphate for further PHB production. Reactions were performed in triplicate (n = 3 biologically independent samples) and data are presented as mean values ± SD. Source data are provided as a Source Data file.

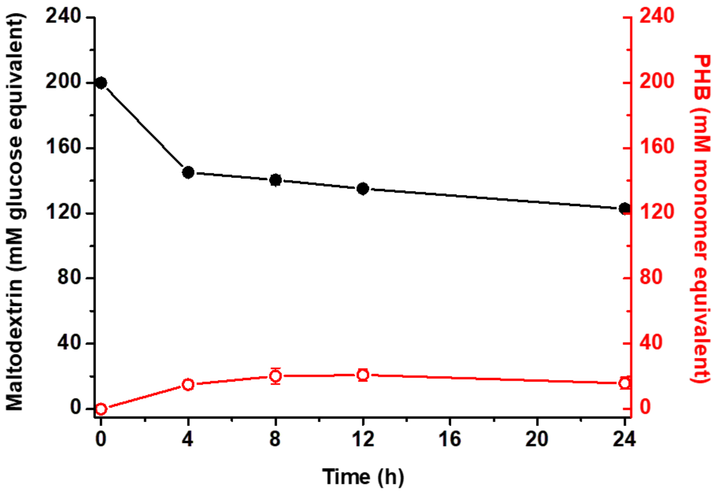

Supplementary Fig. 14 | Production of PHB from 200 mM glucose equivalent of maltodextrin without doubling the enzyme concentrations. Concentrations of enzymes, cofactors, and phosphate ions remained the same as those used for 100 mM substrate in **Fig. 5a**. Reactions were performed in triplicate (n = 3 biologically independent samples) and data are presented as mean values ± SD. Source data are provided as a Source Data file.

Supplementary Table 1. Information of enzymes used in the designed ivSEB

Enzyme	EC number	Source	Specific activity at 37 °C (U/mg)	Reference for specific activity
α-Glucan phosphorylase (αGP)	2.4.1.1	*Escherichia coli*	1.0	1
Phosphoglucomutase (PGM)	5.4.2.2	*Chlostridium thermocellum*	44.6	1
Phosphoglucose isomerase (PGI)	5.3.1.9	*C. thermocellum*	884.7	1
Phosphoketolase (PKL)	4.1.2.9 (XPK); 4.1.2.22 (FPK)	*Bifidobacterium adolescentis*	11.59 ± 0.09 (XPK); 1.38 ± 0.10 (FPK)	This study
Phosphate acetyltransferase (PTA)	2.3.1.8	*E. coli*	20.46 ± 0.23	This study
Acetyl-CoA acetyltransferase (PhaA)	2.3.1.9	*Cupriavidus necator* H16	0.78 ± 0.09[a]	This study
Acetoacetyl-CoA reductase (PhaB)	1.1.1.36	*C. necator* H16	2.06 ± 0.01	This study
Type I polyhydroxybutyrate synthase (PhaC)	2.3.1.304	*Cupriavidus sp.* S-6	8.5[b]	2
Glucose 6-phosphate dehydrogenase (G6PDH)	1.1.1.49	*Thermotoga maritima* MSB8	9.48 ± 0.16	This study
6-phosphogluconate dehydrogenase (6PGDH)	1.1.1.44	*T. maritima* MSB8	2.95 ± 0.01	This study
Transaldolase (TAL)	2.2.1.2	*E. coli*	60[b]	3
Transketolase (TK)	2.2.1.1	*E. coli*	50.4[c]	4
Ribose 5-phosphate isomerase (RPI)	5.3.1.6	*E. coli*	785[b]	3
Ribulose 5-phosphate 3-epimerase (RPE)	5.1.3.1	*E. coli*	729[b]	3
Triose phosphate isomerase (TIM)	5.3.1.1	*Thermus thermophilus* HB27	180	5
Fructose-bisphosphate aldolase (ALD)	4.1.2.13	*E. coli*	477[d]	6
Fructose 1,6-bisphosphatase (FBP)	3.1.3.11	*E. coli*	24.2[b]	7
4-α-Glucanotransferase (4GT)	2.4.1.25	*Thermococcus litoralis* DSM 5473	0.3	1
Polyphosphate glucokinase (PPGK)	2.7.1.63	*Thermobifida fusca* YX[e]	96.4	1

[a] Unit is mU/mg.

[b] Activity was measured at 30 °C.

[c] Activity was measured at 30 °C using Xu5P and R5P as substrates.

[d] Activity was measured at 30 °C using F1,6P as substrate.

[e] PPGK used in this study was a mutant (mutant 4-1) constructed by Zhou *et al.*[8].

Supplementary Table 2. Equilibrium constant (k_{eq}) and Gibbs free energy change ($\Delta_rG'^{\circ}$) values of each enzymatic reaction in the designed ivSEB

Enzyme	Reaction equation for calculation[a]	k_{eq}[b]	$\Delta_rG'^{\circ}$ (kJ/mol)[b]
αGP	Maltotetraose + P_i = G1P + maltotriose[c]	0.1	5.8 ± 3.7
PGM	G1P = G6P	20.0	-7.5 ± 1.5
PGI	G6P = F6P	0.7	1.0 ± 0.8
XPK	Xu5P + P_i = G3P + AcP + H_2O	9.5 x 10^9	-56.9 ± 3.1
FPK	F6P + P_i = E4P + AcP + H_2O	6.6 x 10^7	-44.6 ± 4.0
PTA	AcP + CoA = AcCoA + P_i	180.0	-12.9 ± 1.2
PhaA	2 AcCoA = AcAcCoA + CoA	2.5 x 10^{-5}	26.3 ± 1.7
PhaB	AcAcCoA + NADPH = 3HBCoA + $NADP^+$	110.0	-11.6 ± 7.1
PhaC	3HBCoA → PHB + CoA	Cannot calculate	Cannot calculate
G6PDH	G6P + $NADP^+$ + H_2O = NADPH + 6PG [d]	3.6 x 10^5	-31.7 ± 2.0
6PGDH	6PG + $NADP^+$ + H_2O = NADPH + Ru5P + CO_2 (total)	0.3	2.8 ± 1.8
TAL	F6P + E4P = G3P +S7P	4.0	-3.4 ± 3.9
TK-1	G3P + S7P = Xu5P + R5P	0.3	3.3 ± 3.8
TK-2	Xu5P + E4P = G3P + F6P	140.0	-12.3 ± 3.8
RPI	R5P = Ru5P	0.4	2.6 ± 1.5
RPE	Ru5P = Xu5P	4.0	-3.4 ± 2.3
TIM	G3P = DHAP	10.0	-6.0 ± 1.1
ALD	G3P + DHAP = F1,6P	4.8 x 10^4	-26.7 ± 1.0
FBP	F1,6P + H_2O = F6P + P_i	150.0	-12.4 ± 1.4

[a] For COPASI model construction, water (H_2O) and carbon dioxide (CO_2) in these equations were omitted to simplify the models (see **Supplementary Table 4**).

[b] k_{eq} and $\Delta_rG'^{\circ}$ were calculated by eQuilibrator 3.0 (https://equilibrator.weizmann.ac.il/) under conditions of pH = 7.4, pMg = 2.0, ionic strength = 0.2 M. k_{eq} values were used for the construction of a semi-quantitative computational model by COPASI.

[c] This reaction equation was only used for a rough estimation of k_{eq} and $\Delta_rG'^{\circ}$ of αGP-catalyzed reaction. The actual reaction equation of αGP is written as $(C_6H_{10}O_5)_n + P_i = G1P + (C_6H_{10}O_5)_{n-1}$, which was used for COPASI model construction.

[d] This equation is actually a combination of the G6PDH-catalyzed reaction (G6P + $NADP^+$ = NADPH + 6-phospho-glucono-1,5-lactone) and the subsequent spontaneous hydrolysis of 6-phospho-glucono-1,5-lactone (6PGL; 6PGL + H_2O = 6PG). The self-hydrolysis of 6PGL is an energy-favorable reaction, with k_{eq} and $\Delta_rG'^{\circ}$ values of 2.7 x 10^4 and -25.3 ± 3.3 kJ/mol, respectively. To simplify our COPASI model, it was assumed that all 6PGL produced was converted to 6PG in the designed ivSEB.

Supplementary Table 3. Kinetic functions for COPASI modeling

Number	General reaction formula	Kinetic function	Enzymes to which the function applies
1	A + B = P + Q	$V = \frac{\frac{V_{max}\cdot(A\cdot B-\frac{P\cdot Q}{k_{eq}})}{K_A\cdot K_B}}{\left(1+\frac{A}{K_A}\right)\cdot\left(1+\frac{B}{K_B}\right)+\left(1+\frac{P}{K_P}\right)\cdot\left(1+\frac{Q}{K_Q}\right)-1}$	αGP, PKL, PTA, PhaB, G6PDH, 6PGDH, TAL, TK
2	A = P	$V = \frac{\frac{V_{max}\cdot(A-\frac{P}{k_{eq}})}{K_A}}{\left(1+\frac{A}{K_A}\right)+\left(1+\frac{P}{K_P}\right)-1}$	PGM, PGI, RPI, RPE, TIM
3	2 A = P + Q	$V = \frac{\frac{V_{max}\cdot(A^2-\frac{P\cdot Q}{k_{eq}})}{{K_A}^2}}{(1+\frac{A}{K_A})^2+\left(1+\frac{P}{K_P}\right)\cdot\left(1+\frac{Q}{K_Q}\right)-1}$	PhaA
4	A + B = P	$V = \frac{\frac{V_{max}\cdot(A\cdot B-\frac{P}{k_{eq}})}{K_A\cdot K_B}}{\left(1+\frac{A}{K_A}\right)\cdot\left(1+\frac{B}{K_B}\right)+\left(1+\frac{P}{K_P}\right)-1}$	ALD
5	A = P + Q	$V = \frac{\frac{V_{max}\cdot(A-\frac{P\cdot Q}{k_{eq}})}{K_A}}{\left(1+\frac{A}{K_A}\right)+\left(1+\frac{P}{K_P}\right)\cdot\left(1+\frac{Q}{K_Q}\right)-1}$	FBP
6	A → P + Q	$V = \frac{V_{max}\cdot A}{K_A+A}$	PhaC (for stoichiometric analysis)
7	A → P↓ + Q	$V = \frac{V_{max}\cdot A}{K_A+A}\cdot(1-\frac{C_{bind}\cdot P^n}{V_{max}})$ $= \frac{(V_{max}-P^n\cdot C_{bind})\cdot A}{K_A+A}$	PhaC (for semiquantitative studies)

Supplementary Table 4. Stoichiometric coefficients of enzymatic reactions of the designed ivSEB

Enzyme	Reaction equation for COPASI modeling	Stoichiometric coefficient	
		XPK pathway	FPK pathway
αGP	$(C_6H_{10}O_5)_n + P_i = G1P + (C_6H_{10}O_5)_{n-1}$	3	3
PGM	G1P = G6P	3	3
PGI	G6P = F6P	1	1
XPK	Xu5P + P_i = G3P + AcP	8	0
FPK	F6P + P_i = E4P + AcP	0	8
PTA	AcP + CoA = Ac-CoA + P_i	8	8
PhaA	2 Ac-CoA = AcAc-CoA + CoA	4	4
PhaB	AcAc-CoA + NADPH = 3HB-CoA + $NADP^+$	4	4
PhaC	3HB-CoA → PHB + CoA	4	4
G6PDH	G6P + $NADP^+$ = NADPH + 6PG	2	2
6PGDH	6PG + $NADP^+$ = NADPH + Ru5P	2	2
TAL	F6P + E4P = G3P +S7P	2	2
TK-1	G3P + S7P = Xu5P + R5P	2	2
TK-2	Xu5P + E4P = G3P + F6P	-2[a]	6
RPI	R5P = Ru5P	2	2
RPE	Ru5P = Xu5P	4	4
TIM	G3P = DHAP	3	3
ALD	G3P + DHAP = F1,6P	3	3
FBP	F1,6P = F6P + P_i	3	3

[a] A negative stoichiometric coefficient suggests the net reaction flows towards the reverse direction.

Supplementary Table 5. Kinetic parameters for COPASI modeling

Enzyme	Parameter	Parameter value[a] Model 0 (before fitting)[b]	Model 1 (after 1st round of fitting)	Model 2 (after 2nd round of fitting)
	$K_{m,\ \alpha GP,\ (C_6H_{10}O_5)_n}$	0.2 mM[9]		
	$K_{m,\ \alpha GP,\ Pi}$	0.5 mM[9]		Same as model 1
αGP	$K_{m,\ \alpha GP,\ G1P}$	1 mM[9]	Same as model 0	
	$K_{m,\ \alpha GP,\ (C_6H_{10}O_5)_{n-1}}$	3.6 mM[9]		
	$k_{eq,\ \alpha GP}$	0.1		0.004 (↓)
	$K_{m,\ PGM,\ G1P}$	0.41 mM[10]		
PGM	$K_{m,\ PGM,\ G6P}$	13 mM[11]	Same as model 0	Same as model 1
	$k_{eq,\ PGM}$	20.0		
	$K_{m,\ PGI,\ G6P}$	0.3 mM[12]		
PGI	$K_{m,\ PGI,\ F6P}$	0.15 mM[12]	Same as model 0	Same as model 1
	$k_{eq,\ PGI}$	0.7		
	$K_{m,\ PKL,\ Xu5P}$	3.6 mM[13]	0.2 mM (↓)	
	$K_{m,\ PKL,\ F6P}$	9.96 mM[14]	2 mM (↓)	
	$K_{m,\ PKL,\ Pi}$	1.2 mM[15]	0.05 mM (↓)	
PKL	$K_{m,\ PKL,\ G3P}$	0.1 mM		Same as model 1
	$K_{m,\ PKL,\ E4P}$	0.1 mM		
	$K_{m,\ PKL,\ AcP}$	0.1 mM	Same as model 0	
	$k_{eq,\ XPK}$	9.5×10^{9}		
	$k_{eq,\ FPK}$	6.6×10^{7}		
	$K_{m,\ PTA,\ AcP}$	0.9 mM[16]	0.02 mM (↓)	
	$K_{m,\ PTA,\ CoA}$	0.067 mM[16]	0.01 mM (↓)	
PTA	$K_{m,\ PTA,\ AcCoA}$	0.045 mM[16]		Same as model 1
	$K_{m,\ PTA,\ Pi}$	2.1 mM[16]	Same as model 0	
	$k_{eq,\ PTA}$	180.0		
	$K_{m,\ PhaA,\ AcCoA}$	0.0258 mM[17]		
PhaA	$K_{m,\ PhaA,\ AcAcCoA}$	0.1 mM[12]	Same as model 0	Same as model 1
	$K_{m,\ PhaA,\ CoA}$	0.1 mM[12]		
	$k_{eq,\ PhaA}$	2.5×10^{-5}		
	$K_{m,\ PhaB,\ AcAcCoA}$	0.005 mM[18]	0.001 mM (↓)	
	$K_{m,\ PhaB,\ NADPH}$	0.019 mM[18]	0.005 mM (↓)	
PhaB	$K_{m,\ PhaB,\ 3HBCoA}$	0.033 mM[18]		Same as model 1
	$K_{m,\ PhaB,\ NADP+}$	0.031 mM[18]	Same as model 0	
	$k_{eq,\ PhaB}$	110.0		
	$K_{m,\ PhaC,\ 3HBCoA}$	0.19 mM[19]	Same as model 0	Same as model 1
PhaC	C_{bind}	1000	830 (↓)	770 (↓)
	n	1	2 (↑)	Same as model 1

Enzyme	Parameter	Parameter value[a] Model 0 (before fitting)[b]	Model 1 (after 1st round of fitting)	Model 2 (after 2nd round of fitting)
G6PDH	$K_{m, G6PDH, G6P}$	0.07 mM[20]	Same as model 0	Same as model 1
	$K_{m, G6PDH, NADP+}$	0.0246 mM[20]		
	$K_{m, G6PDH, NADPH}$	0.023 mM[21]		
	$K_{m, G6PDH, 6PG}$	0.279 mM[21]		
	$k_{eq, G6PDH}$	3.6×10^{5}		
6PGDH	$K_{m, 6PGDH, 6PG}$	0.011 mM[22]	Same as model 0	Same as model 1
	$K_{m, 6PGDH, NADP+}$	0.01 mM[22]		
	$K_{m, 6PGDH, NADPH}$	0.002 mM[23]		
	$K_{m, 6PGDH, Ru5P}$	1 mM[23]		
	$k_{eq, 6PGDH}$	0.3		
TAL	$K_{m, TAL, F6P}$	1.2 mM[24]	Same as model 0	Same as model 1
	$K_{m, TAL, E4P}$	0.09 mM[24]		
	$K_{m, TAL, G3P}$	0.038 mM[24]		
	$K_{m, TAL, S7P}$	0.285 mM[24]		
	$k_{eq, TAL}$	4.0		
TK	$K_{m, TK, G3P}$	2.1 mM[4]	0.1 mM (↓)	Same as model 1
	$K_{m, TK, S7P}$	4 mM[4]	0.1 mM (↓)	
	$K_{m, TK, Xu5P}$	0.16 mM[4]	Same as model 0	
	$K_{m, TK, R5P}$	1.4 mM[4]		
	$K_{m, TK, E4P}$	0.09 mM[4]		
	$K_{m, TK, F6P}$	1.1 mM[4]		
	$k_{eq, TK-1}$	0.3		
	$k_{eq, TK-2}$	140.0		
RPI	$K_{m, RPI, R5P}$	0.108 mM[25]	Same as model 0	Same as model 1
	$K_{m, RPI, Ru5P}$	0.1 mM		
	$k_{eq, RPI}$	0.4		
RPE	$K_{m, RPI, Ru5P}$	0.056 mM[26]	Same as model 0	Same as model 1
	$K_{m, RPI, Xu5P}$	0.1 mM		
	$k_{eq, RPE}$	4.0		
TIM	$K_{m, TIM, G3P}$	1 mM[12]	Same as model 0	Same as model 1
	$K_{m, TIM, DHAP}$	1 mM[12]		
	$k_{eq, TIM}$	10.0		
ALD	$K_{m, ALD, G3P}$	0.1 mM[12]	Same as model 0	Same as model 1
	$K_{m, ALD, DHAP}$	0.1 mM[12]		
	$K_{m, ALD, F1,6P}$	0.015 mM[12]		
	$k_{eq, ALD}$	4.8×10^{4}		

Enzyme	Parameter	Parameter value[a] Model 0 (before fitting)[b]	Model 1 (after 1st round of fitting)	Model 2 (after 2nd round of fitting)
FBP	$K_{m,\ FBP,\ F1,6P}$	0.016 mM[27]	Same as model 0	Same as model 1
	$K_{m,\ FBP,\ F6P}$	0.1 mM		
	$K_{m,\ FBP,\ Pi}$	0.1 mM		
	$k_{eq,\ FBP}$	150.0		

[a] Each parameter adjusted during model fitting was annotated with a bracket featuring an upward or downward arrow indicating its up- or downregulation.

[b] For model 0, K_m values that could not be found in databases were set as 0.1 mM. k_{eq} values were calculated by eQuilibrator 3.0 (https://equilibrator.weizmann.ac.il/) under conditions of pH = 7.4, pMg = 2.0, ionic strength = 0.2 M. C_{bind} and n were randomly set as 1000 and 1, respectively.

Supplementary Table 6. Comparison of enzyme loading amounts prior and after in silico optimization[a]

Content	Before simulation optimization[b]		After first round of in silico optimization using Model 1[c]		After second round of in silico optimization using Model 2[d]	
	U/mL	mg/mL	U/mL	mg/mL	U/mL	mg/mL
αGP	5	5.00	2	2.00	3	3.00
PGM	5	0.11	3	0.07	5	0.11
PGI	5	0.006	4	0.005	3	0.003
PKL	5[e]	0.43	5[e]	0.43	5[e]	0.43
PTA	5	0.24	4	0.20	3	0.15
PhaA	3.9×10^{-3}	5.00	1.56×10^{-3}	2.00	1.56×10^{-3}	2.00
PhaB	5	2.43	5	2.43	4	1.94
PhaC	5	0.59	8	0.94	8	0.94
TAL	5	0.08	5	0.08	5	0.08
TK	5[f]	0.10	4[f]	0.08	3[f]	0.06
RPI	5	0.006	3	0.004	1	0.001
RPE	5	0.007	5	0.007	4	0.005
TIM	5	0.03	2	0.01	1	0.006
ALD	5	0.01	2	0.004	2	0.004
FBP	5	0.21	3	0.12	5	0.21
G6PDH	5	0.53	1	0.11	1	0.11
6PGDH	5	1.69	3	1.01	1	0.34
Total	-	16.47	-	9.50	-	9.39

[a] For PhaA, concentrations in U/mL were calculated from the values in mg/mL and the specific activity of PhaA. For the rest of enzymes, concentrations in mg/mL were calculated from values in U/mL and the specific activities. Specific activities of enzymes are listed in **Supplementary Table 1**.

[b] This set of enzyme loading concentrations correlates to **Fig. 4b**.

[c] This set of enzyme loading concentrations correlates to **Fig. 4c**.

[d] This set of enzyme loading concentrations correlates to **Fig. 4d**.

[e] In terms of XPK activity.

[f] In terms of TK-1 activity.

Supplementary Table 7. Molecular weights of PHB samples

Number	Source	M_W (× 10^5)	M_n (× 10^5)	PDI (i.e. M_W/M_n)
**1**	Natural origin (purchased from Sigma-Aldrich)	4.08	2.07	1.97
**2**	Produced by engineered microbes	4.25	2.23	1.90
**3**	Produced by using our ivSEB	2.97	1.80	1.65

Supplementary Table 8. Primers used for plasmid construction

Primers	Sequence (5' → 3')
**PhaA-IF**	CCTGGTGCCGCGCGGCAGCCATATGATGACTGACGTTGTCATCGTATCCG
**PhaA-IR**	GTGGTGGTGGTGGTGGTGCTCGAGTTATTTGCGCTCGACTGCCAGCGCC
**PhaA-VF**	GGCGCTGGCAGTCGAGCGCAAATAACTCGAGCACCACCACCACCACCAC
**PhaA-VR**	CGGATACGATGACAACGTCAGTCATCATATGGCTGCCGCGCGGCACCAGG
**PhaB-IF**	GTGGACAGCAAATGGGTCGCGGATCCATGACTCAGCGCATTGCGTATGTGAC
**PhaB-IR**	CAGTGGTGGTGGTGGTGGTGCTCGAGTCAGCCCATATGCAGGCCGCCGTTG
**PhaB-VF**	CAACGGCGGCCTGCATATGGGCTGACTCGAGCACCACCACCACCACCACTG
**PhaB-VR**	GTCACATACGCAATGCGCTGAGTCATGGATCCGCGACCCATTTGCTGTCCAC

Supplementary References

1. Wei, X., Li, Q., Hu, C. & You, C. An ATP-free in vitro synthetic enzymatic biosystem facilitating one-pot stoichiometric conversion of starch to mannitol. *Appl. Microbiol. Biotechnol.* **105**, 1913-1924 (2021).
2. Song, J. J., Zhang, S., Lenz, R. W. & Goodwin, S. In vitro polymerization and copolymerization of 3-hydroxypropionyl-CoA with the PHB synthase from Ralstonia eutropha. *Biomacromolecules.* **1**, 433-439 (2000).
3. Chen, Y. et al. A computationally designed pathway for one carbon compounds utilization and its in vitro construction. *Chin. J. Bioprocess Eng.* **15**, 86-92 (2017).
4. Sprenger, G. A., Schorken, U., Sprenger, G. & Sahm, H. Transketolase A of *Escherichia coli* K12. Purification and properties of the enzyme from recombinant strains. *Eur. J. Biochem.* **230**, 525-532 (1995).
5. Myung, S. et al. In vitro metabolic engineering of hydrogen production at theoretical yield from sucrose. *Metab. Eng.* **24**, 70-77 (2014).
6. Baldwin, S. A., Perham, R. N. & Stribling, D. Purification and characterization of the class-II D-fructose 1,6-bisphosphate aldolase from *Escherichia coli* (Crookes' strain). *Biochem. J.* **169**, 633-641 (1978).
7. Kelley-Loughnane, N. et al. Purification, kinetic studies, and homology model of *Escherichia coli* fructose-1,6-bisphosphatase. *Biochim. Biophys. Acta.* **1594**, 6-16 (2002).
8. Zhou, W., Huang, R., Zhu, Z. G. & Zhang, Y.-H. P. J. Coevolution of both thermostability and activity of polyphosphate glucokinase from *Thermobifida fusca* YX. *Appl. Environ. Microbiol.* **84**, e01224-01218 (2018).
9. Boeck, B. & Schinzel, R. Purification and characterisation of an alpha-glucan phosphorylase from the thermophilic bacterium *Thermus thermophilus*. *Eur. J. Biochem.* **239**, 150-155 (1996).
10. Wang, Y. & Zhang, Y. H. A highly active phosphoglucomutase from *Clostridium thermocellum*: cloning, purification, characterization and enhanced thermostability. *J. Appl. Microbiol.* **108**, 39-46 (2010).
11. Ray, W. K. et al. A phosphohexomutase from the archaeon *Sulfolobus solfataricus* is covalently modified by phosphorylation on serine. *J. Bacteriol.* **187**, 4270-4275 (2005).
12. Korman, T. P., Opgenorth, P. H. & Bowie, J. U. A synthetic biochemistry platform for cell free production of monoterpenes from glucose. *Nat. Commun.* **8**, 15526 (2017).
13. Yevenes, A. & Frey, P. A. Cloning, expression, purification, cofactor requirements, and steady state kinetics of phosphoketolase-2 from *Lactobacillus plantarum*. *Bioorg. Chem.* **36**, 121-127 (2008).
14. Dele-Osibanjo, T. et al. Growth-coupled evolution of phosphoketolase to improve L-glutamate production by *Corynebacterium glutamicum*. *Appl. Microbiol. Biotechnol.* **103**, 8413-8425 (2019).
15. Suzuki, R. et al. Crystal structures of phosphoketolase: thiamine diphosphate-dependent dehydration mechanism. *J. Biol. Chem.* **285**, 34279-34287 (2010).

16. Campos-Bermudez, V. A., Bologna, F. P., Andreo, C. S. & Drincovich, M. F. Functional dissection of *Escherichia coli* phosphotransacetylase structural domains and analysis of key compounds involved in activity regulation. *FEBS J.* **277**, 1957-1966 (2010).
17. Vishwakarma, R. K. et al. Molecular cloning, biochemical characterization, and differential expression of an Acetyl-coA C-ccetyltransferase gene (AACT) of brahmi (*Bacopa monniera*). *Plant Mol. Biol. Rep.* **31**, 547-557 (2013).
18. Belova, L. L., Sokolov, A. P., Sidorov, I. A. & Trotsenko, Y. A. Purification and characterization of NADPH-dependent acetoacetyl-CoA reductase from *Methylobacterium extorquens*. *FEMS Microbiol. Lett.* **156**, 275-279 (1997).
19. Normi, Y. M. et al. Characterization and properties of G4X mutants of *Ralstonia eutropha* PHA synthase for poly(3-hydroxybutyrate) biosynthesis in *Escherichia coli*. *Macromol. Biosci.* **5**, 197-206 (2005).
20. Chassagnole, C. et al. Dynamic modeling of the central carbon metabolism of Escherichia coli. *Biotechnol. Bioeng.* **79**, 53-73 (2002).
21. Cho, S. W. & Joshi, J. G. Characterization of glucose-6-phosphate dehydrogenase isozymes from human and pig brain. *Neuroscience.* **38**, 819-828 (1990).
22. Wang, Y. & Zhang, Y. H. Overexpression and simple purification of the *Thermotoga maritima* 6-phosphogluconate dehydrogenase in *Escherichia coli* and its application for NADPH regeneration. *Microb. Cell Fact.* **8**, 30 (2009).
23. Berdis, A. J. & Cook, P. F. Overall kinetic mechanism of 6-phosphogluconate dehydrogenase from *Candida utilis*. *Biochemistry.* **32**, 2036-2040 (1993).
24. Sprenger, G. A., Schorken, U., Sprenger, G. & Sahm, H. Transaldolase B of *Escherichia coli* K-12: cloning of its gene, *talB*, and characterization of the enzyme from recombinant strains. *J. Bacteriol.* **177**, 5930-5936 (1995).
25. Horitsu, H. et al. Purification, properties and structure of ribose 5-phosphate ketol isomerase from *Candida utilis*. *Agric. Biol. Chem.* **40**, 257-264 (1976).
26. Le, S. B. et al. 6-Phosphofructokinase and ribulose-5-phosphate 3-epimerase in methylotrophic *Bacillus methanolicus* ribulose monophosphate cycle. *Appl. Microbiol. Biotechnol.* **101**, 4185-4200 (2017).
27. Hines, J. K., Fromm, H. J. & Honzatko, R. B. Structures of activated fructose-1,6-bisphosphatase from *Escherichia coli*. Coordinate regulation of bacterial metabolism and the conservation of the R-state. *J. Biol. Chem.* **282**, 11696-11704 (2007).

Source Data file: Supplementary Fig. 1

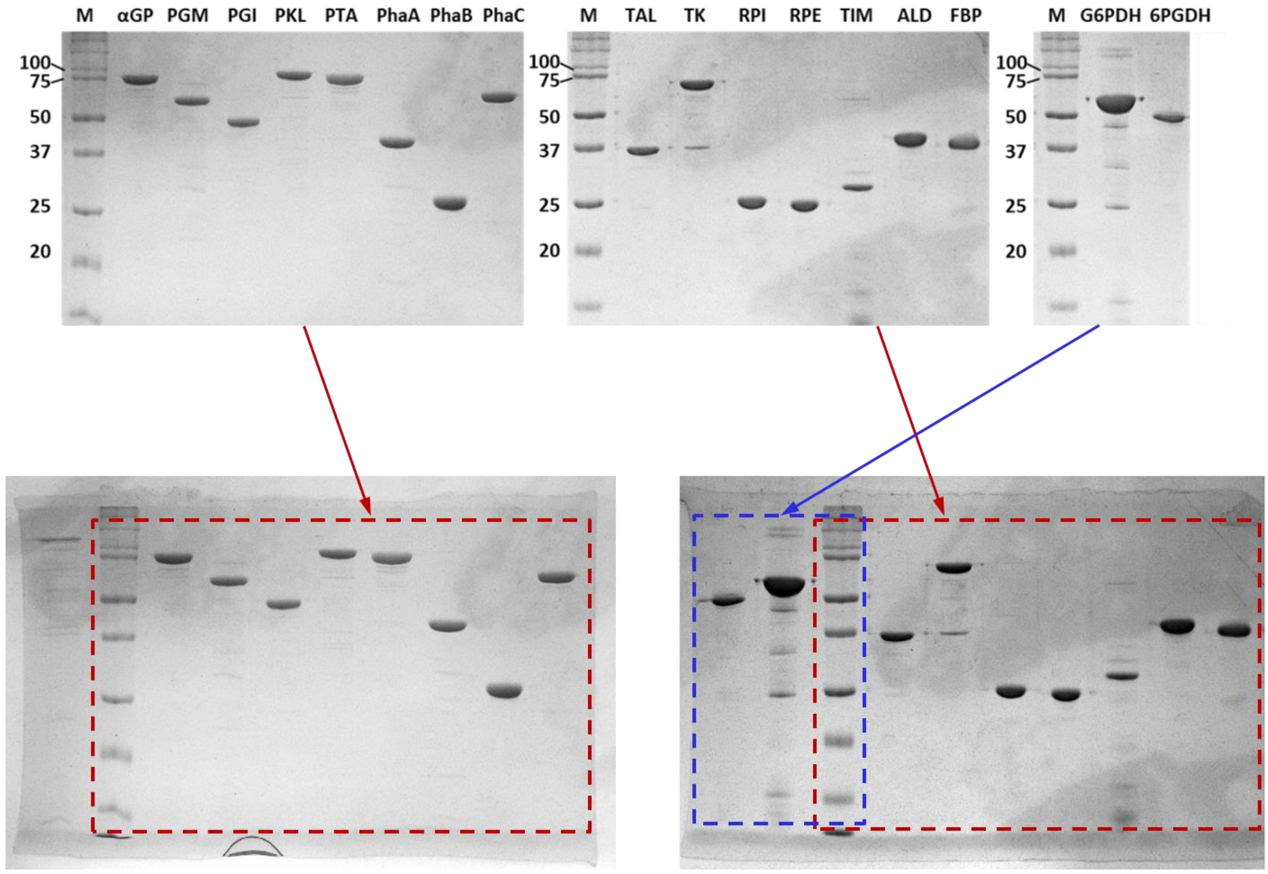

Supplement: Supplementary file 1 — Supplementary Information [file 41467_2024_46871_MOESM1_ESM.pdf]
