# Supplementary material for: ATP-free in vitro biotransformation of starch-derived maltodextrin into poly-3-hydroxybutyrate via acetyl-CoA
Source: Nat Commun. 2024 Apr 16;15:3267. doi: 10.1038/s41467-024-46871-y (PMC11021460; doi:10.1038/s41467-024-46871-y)
Supplement: Supplementary file 3 — Reporting Summary [file 41467_2024_46871_MOESM3_ESM.pdf]

nature portfolio

Corresponding author(s): Chun You

Last updated by author(s): Mar 4, 2024

Reporting Summary

Nature Portfolio wishes to improve the reproducibility of the work that we publish. This form provides structure for consistency and transparency in reporting. For further information on Nature Portfolio policies, see our Editorial Policies and the Editorial Policy Checklist.

Statistics

For all statistical analyses, confirm that the following items are present in the figure legend, table legend, main text, or Methods section.

n/a	Confirmed	
☐	☒	The exact sample size (n) for each experimental group/condition, given as a discrete number and unit of measurement
☐	☒	A statement on whether measurements were taken from distinct samples or whether the same sample was measured repeatedly
☒	☐	The statistical test(s) used AND whether they are one- or two-sided<br>*Only common tests should be described solely by name; describe more complex techniques in the Methods section.*
☒	☐	A description of all covariates tested
☒	☐	A description of any assumptions or corrections, such as tests of normality and adjustment for multiple comparisons
☐	☒	A full description of the statistical parameters including central tendency (e.g. means) or other basic estimates (e.g. regression coefficient) AND variation (e.g. standard deviation) or associated estimates of uncertainty (e.g. confidence intervals)
☒	☐	For null hypothesis testing, the test statistic (e.g. F, t, r) with confidence intervals, effect sizes, degrees of freedom and P value noted<br>*Give P values as exact values whenever suitable.*
☒	☐	For Bayesian analysis, information on the choice of priors and Markov chain Monte Carlo settings
☒	☐	For hierarchical and complex designs, identification of the appropriate level for tests and full reporting of outcomes
☒	☐	Estimates of effect sizes (e.g. Cohen's d, Pearson's r), indicating how they were calculated

Our web collection on statistics for biologists contains articles on many of the points above.

Software and code

Policy information about availability of computer code

Data collection	Cary WinUV (version 4.20) was used to collect the absorbance data; Image Lab Software (version 3.0) was used to collect the SDS-PAGE results.
Data analysis	CopasiUI (version 4) was used to construct the computational model, collect and analyze the simulation data. D7900P software (Techcomp, China) was used to collect and analyze the gas chromatographic data. CirrusTM GPC software (version 3.4) was used to collect and analyze the molecular weight data. Other data was analyzed using Microsoft Excel 2019 or OriginPro (version 8). Reaction pathways were drawn using ChemBioDraw (version 14.0). Multi-panel Figures were arranged using Adobe Illustrator CC 2014.

For manuscripts utilizing custom algorithms or software that are central to the research but not yet described in published literature, software must be made available to editors and reviewers. We strongly encourage code deposition in a community repository (e.g. GitHub). See the Nature Portfolio guidelines for submitting code & software for further information.

Data

Policy information about availability of data

All manuscripts must include a data availability statement. This statement should provide the following information, where applicable:

- Accession codes, unique identifiers, or web links for publicly available datasets
- A description of any restrictions on data availability
- For clinical datasets or third party data, please ensure that the statement adheres to our policy

Databases used in the study are: BRENDA database (www.brenda-enzymes.org/), eQuilibrator (https://equilibrator.weizmann.ac.il/). Data supporting the findings of

this study are available within the article and its Supplementary Information. The source data underlying Figure 3c, Figure 4, Figure 5, Supplementary Figure 2, Supplementary Figure 3a-3d, Supplementary Figures 4-10, Supplementary Figure 11b and 11c, and Supplementary Figures 12-14 are provided as a Source Data file. Source data are provided with this paper.

Research involving human participants, their data, or biological material

Policy information about studies with human participants or human data. See also policy information about sex, gender (identity/presentation), and sexual orientation and race, ethnicity and racism.

Reporting on sex and gender	N/A
Reporting on race, ethnicity, or other socially relevant groupings	N/A
Population characteristics	N/A
Recruitment	N/A
Ethics oversight	N/A

Note that full information on the approval of the study protocol must also be provided in the manuscript.

Field-specific reporting

Please select the one below that is the best fit for your research. If you are not sure, read the appropriate sections before making your selection.

- [x] Life sciences
- [ ] Behavioural & social sciences
- [ ] Ecological, evolutionary & environmental sciences

For a reference copy of the document with all sections, see nature.com/documents/nr-reporting-summary-flat.pdf

Life sciences study design

All studies must disclose on these points even when the disclosure is negative.

Sample size	The sample size is reported in the manuscript and in the legend of each figure. No statistical method was used to determine sample size. We referred to the design in most published studies that if 3 replicates exhibit sufficient reproducibility, experiments were considered as reproducible and completed.
Data exclusions	No data were excluded from the analyses.
Replication	Three independent replicates were performed for all experiments at least. All attempts at replication were successful.
Randomization	Randomization was not relevant to this study since we did not have any participants and medical or environmental samples in our study.
Blinding	Blinding was not relevant to this study because there was no individual/subjective bias in experimentation or data analysis.

Reporting for specific materials, systems and methods

We require information from authors about some types of materials, experimental systems and methods used in many studies. Here, indicate whether each material, system or method listed is relevant to your study. If you are not sure if a list item applies to your research, read the appropriate section before selecting a response.

Materials & experimental systems

n/a	Involved in the study	
x		Antibodies
x		Eukaryotic cell lines
x		Palaeontology and archaeology
x		Animals and other organisms
x		Clinical data
x		Dual use research of concern
x		Plants

Methods

n/a	Involved in the study	
x		ChIP-seq
x		Flow cytometry
x		MRI-based neuroimaging

Plants

Seed stocks	N/A
Novel plant genotypes	N/A
Authentication	N/A
